# Efficacy of docetaxel combined carboplatin for the treatment of patients with castration-resistant prostate cancer: A protocol of systematic review and meta-analysis

**DOI:** 10.1097/MD.0000000000020297

**Published:** 2020-05-22

**Authors:** Chun-Lin Pu, Jiu-Zhi Li, Wen-Long Fan

**Affiliations:** a Department of Urology Center, People's Hospital of Xinjiang Uygur Antonomous Region; b Department of Urology Center, Xinjiang Medical University, Urumqi, Xinjiang, China.

**Keywords:** carboplatin, castration-resistant prostate cancer, docetaxel, efficacy, safety

## Abstract

**Background:**

A numerous published studies have reported that docetaxel combined carboplatin (DC) has been utilized for the treatment of patients with castration-resistant prostate cancer (CRPC). However, there are still contradictory results. Therefore, this systematic review and meta-analysis will explore the efficacy and safety of DC for the treatment of patients with CRPC.

**Methods:**

We will systematically and comprehensively search MEDLINE, EMBASE, Cochrane Library, Web of Science, CINAHL, WANGFANG, CBM, and CNKI from the beginning up to the March 1, 2020, regardless language and publication time. We will consider randomized controlled trials that evaluated the efficacy and safety of DC for the treatment of patients with CRPC. The treatment effects of all dichotomous data will be estimated as risk ratio and 95% confidence intervals (CIs), and that of continuous outcomes will be calculated as standardized mean difference or mean difference and 95% CIs. Methodological quality will be appraised by Cochrane risk of bias tool, and quality of evidence will be identified by Grading of Recommendations Assessment Development and Evaluation. Statistical analysis will be undertaken by RevMan 5.3 software.

**Results:**

This study will systematically explore the efficacy and safety of DC for the treatment of patients with CRPC.

**Conclusion:**

This study may provide helpful evidence to determine whether DC is an effective treatment for patients with CRPC or not.

**Systematic review registration:**

INPLASY202040076.

## 1 Introduction

Castrate-resistant prostate cancer (CRPC) is progressive prostate cancer (PC) metastases in men.^[1–5]^ It is characterized by a continuous rise in serum prostate-specific antigen levels.^[6–10]^ It has been estimated that about 165,000 new PC cases, and 30,000 patients will die from PC.^[11,12]^ Despite most patients with PC are cured, 20% of them may experience recurrence of this disease.^[13]^ Although androgen deprivation therapy is utilized for the treatment of patients with recurrent, progressive, or metastatic PC, most of them will eventually develop CRPC.^[14,15]^

A numerous studies have reported to use docetaxel combined carboplatin (DC) for the treatment of patients with CRPC.^[16–21]^ However, its efficacy and safety is still contrary, and no systematic review specifically has focused on this issue. Thus, this study will evaluate the efficacy and safety of DC for the treatment of patients with CRPC.

## 2 Methods

### 2.1 Study registration

This study has been funded and registered on INPLASY202040076. It has been reported follows the Preferred Reporting Items for Systematic Reviews and Meta-Analysis Protocol statement guidelines.^[22]^

### 2.2 Inclusion criteria

#### 2.2.1 Participants

We will include all male participants who were diagnosed as CRPC, in spite of their country, race, age, and duration and severity of CRPC.

#### 2.2.2 Interventions/exposure

In the experimental group, all patients underwent DC as their treatment for CRPC.

In the control group, there are no restrictions to the control treatments. However, we will exclude studies that utilized DC or any comparators involved DC.

#### 2.2.3 Study types

All potential randomized controlled trials (RCTs) that investigated the efficacy and safety of DC for the treatment of patients with CRPC will be included. However, any other studies, such as animal studies, case studies, reviews, comments, non-clinical studies, and uncontrolled clinical studies will be excluded.

#### 2.2.4 Outcome measurements

##### 2.2.4.1 Primary outcome

Disease-free survival (defined as length of time after treatment during which no disease is found);

##### 2.2.4.2 Secondary outcomes

Overall survival (defined as the time from randomization to death by any reasons);

Progression-free survival (defined as the time from randomization to disease progression or death by any reasons);

Prostate-specific antigen (PSA) response rate;

Duration of PSA response;

Quality of life (as measured by any relevant scales reported in the trials); and adverse events.

### 2.3 Literature search

This review will systematically and comprehensively retrieve all potential RCTs in MEDLINE, EMBASE, Cochrane Library, Web of Science, CINAHL, WANGFANG, CBM, and CNKI from the beginning up to the March 1, 2020, regardless language and publication time. All related RCTs that assessed the efficacy and safety of DC for the treatment of patients with CRPC will be considered for inclusion. We have created search strategy sample for MEDLINE in Table 1, and will adapt similar search strategies for other electronic databases.

**Table 1:**
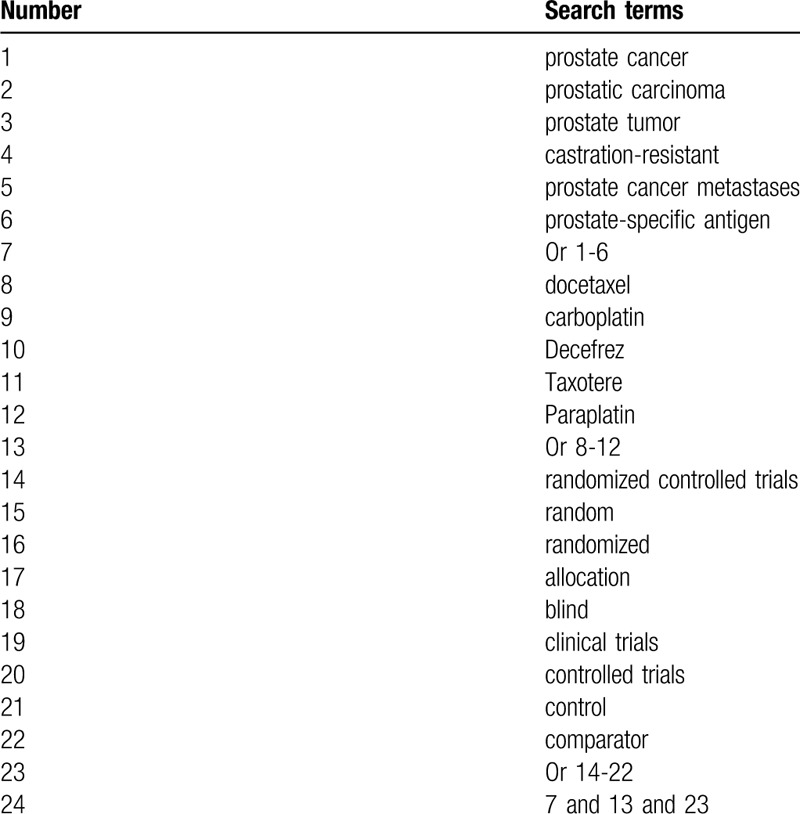
Search strategy sample of MEDLINE.

In addition, we will seek other resources for more potential studies, such as dissertations and reference lists of associated reviews.

### 2.4 Study selection

Two members of our study team will independently scan the titles/abstracts of all searched citations according to the previously designed eligibility criteria. Literatures that are irrelevant to study topic will be excluded. Full papers of all remaining studies will be further read to judge if they fulfill all inclusion criteria. Any disagreements will be solved by discussion with a third team member. The procedure of study selection is demonstrated in a flow chart.

### 2.5 Data extraction and management

Two team members will extract data using a pre-designed data extraction sheet. It comprises of the following information: publication information (such as first author, time of publication), patient characteristics (such as race, age, eligibility criteria, duration, and severity of CRPC), study design (such as sample size, randomization details), details of treatment and comparators, outcome indicators, safety, follow-up data, and conflict of interest. Any differences between 2 members will be solved by consulting a third member and a consensus will be reached after discussion. If insufficient or missing data is examined, we will contact primary authors to request it.

### 2.6 Study quality assessment

Two team members will independently assess methodological quality by Cochrane Collaboration's tool. We will appraise study quality for each eligible trial through seven aspects and each one is graded as low, unclear or high risk of bias. Any different views between two members will be settled by a third member through a consensus meeting.

### 2.7 Measurement of treatment effect

We will estimate the pooled treatment effects for continuous data as mean difference or standardized mean difference and 95% confidence intervals (CIs), and dichotomous data as risk ratio and 95% CIs.

### 2.8 Assessment of heterogeneity

We will employ *I*^*2*^ test to examine statistical heterogeneity among included trials. We define *I*^*2*^ ≤ 50% as homogeneity, and *I*^*2*^ > 50% as obvious heterogeneity.

### 2.9 Data synthesis

We will apply RevMan 5.3 software to analyze outcome data and perform a meta-analysis. If homogeneity is found, a fixed-effects model will be used for data pooling, and a meta-analysis will be carried out if sufficient data is extracted. On the other hand, if considerable heterogeneity is examined, a random-effects model will be utilized for data synthesis, and a subgroup analysis will performed to test the sources of remarkable heterogeneity. If we can still identify obvious heterogeneity after subgroup analysis, we will not consider conducting a meta-analysis.

### 2.10 Subgroup analysis

We will carry out a subgroup analysis to figure out sources of obvious heterogeneity across studies according to the different types of treatments, controls, and outcomes.

### 2.11 Sensitivity analysis

We will conduct a sensitivity analysis to test the robustness of study findings by removing studies with high risk of bias.

### 2.12 Reporting bias

We will undertake a funnel plot and Egger regression test to identify any possible reporting biases if at least 10 RCTs are included.^[23]^

### 2.13 Dissemination and ethics

We will submit this study on an academic journal or a conference meeting. This study will not collect privacy data, since no ethical approval is required.

## 3 Discussion

CRPC is a very tricky type of PC. Although a variety of previous studies have found that DC can be used to treat CRPC, their results are still inconsistent.^[16–21]^ The purpose of this study is to summarize the most recent evidence of DC in the treatment of CRPC. To avoid potential bias, we will collect all relevant records as comprehensive as possible. As to exploration the potential obvious heterogeneity, we will carry out subgroup analysis. The findings of this study will help to determine whether DC is effective and safety for the treatment of patients with CRPC.

## Author contributions

**Conceptualization:** Chun-Lin Pu, Jiu-Zhi Li, Wen-Long Fan.

**Data curation:** Wen-Long Fan.

**Formal analysis:** Chun-Lin Pu.

**Funding acquisition:** Wen-Long Fan.

**Investigation:** Wen-Long Fan.

**Methodology:** Chun-Lin Pu, Jiu-Zhi Li.

**Project administration:** Wen-Long Fan.

**Resources:** Chun-Lin Pu, Jiu-Zhi Li.

**Software:** Chun-Lin Pu, Jiu-Zhi Li.

**Supervision:** Wen-Long Fan.

**Validation:** Chun-Lin Pu, Jiu-Zhi Li, Wen-Long Fan.

**Visualization:** Chun-Lin Pu, Wen-Long Fan.

**Writing – original draft:** Chun-Lin Pu, Jiu-Zhi Li, Wen-Long Fan.

**Writing – review & editing:** Chun-Lin Pu, Jiu-Zhi Li, Wen-Long Fan.
